# Identification and characterization of *in silico*, *in vivo*, *in vitro*, and reactive metabolites of infigratinib using LC-ITMS: bioactivation pathway elucidation and *in silico* toxicity studies of its metabolites†

**DOI:** 10.1039/c9ra10871h

**Published:** 2020-04-23

**Authors:** Nasser S. Al-Shakliah, Mohamed W. Attwa, Adnan A. Kadi, Haitham AlRabiah

**Affiliations:** Department of Pharmaceutical Chemistry, College of Pharmacy, King Saud University P. O. Box 2457 Riyadh 11451 Saudi Arabia mzeidan@ksu.edu.sa +966 1146 76 220 +966 1146 70237; Department of Pharmaceutical Chemistry, College of Pharmacy, Aden University P. O. Box 6312 Yemen; Students' University Hospital, Mansoura University Mansoura 35516 Egypt

## Abstract

Infigratinib (INF) is a novel, small molecule that is orally administered to inhibit human fibroblast growth factor receptors (FGFRs), which are a family of receptor tyrosine kinases that may be upregulated in different tumor cell types. On 6 January 2020, the FDA granted fast track designation to INF for first-line treatment of cholangiocarcinoma. Prediction of susceptible sites of metabolism and reactivity pathways (cyanide and GSH) for INF was performed by the Xenosite web predictor tool. Then, we report the characterization and identification of *in vitro*, *in vivo*, and reactive intermediates of INF using liquid chromatography ion trap mass spectrometry (LC-ITMS). Finally, an *in silico* toxicity assessment of INF metabolites was carried out using the StarDrop DEREK module showing structural alerts. Rat liver microsomes (RLMs) and isolated perfused rat liver hepatocytes were incubated with INF *in vitro* and the generated metabolites were collected by protein precipitation. *In vivo* metabolism was evaluated by time-course urine sampling from Sprague-Dawley rats administered a single INF oral dose. A similar volume of acetonitrile was added to each collected urine sample and both organic and aqueous layers were analyzed by LC-ITMS to detect *in vivo* INF metabolites. *N*-Ethyl piperazine rings and benzene at part A of the INF structure are metabolized to form iminium and 1,4-benzoquinone, respectively, which are very reactive toward nucleophilic macromolecules. Incubation of INF with RLMs in the presence of 1.0 mM KCN and 1.0 mM glutathione was used to evaluate reactive metabolites potentially responsible for toxicities associated with INF. There were seven *in vitro* phase I metabolites, three *in vitro* phase II metabolites, three cyano adducts, and three GSH conjugate metabolites of INF detected by LC-ITMS. *In vivo* INF metabolites identified included four *in vivo* phase I and three *in vivo* phase II metabolites. *In vitro* and *in vivo* phase I metabolic pathways included *N*-dealkylation, *N*-demethylation, *O*-demethylation, hydroxylation, and dechlorination, while the *in vivo* phase II metabolic reaction was a direct conjugation of INF with glucuronic acid and sulphate.

## Abstract 2

An *in silico* web designer tool was utilized to guide laboratory work for infigratinib metabolism. Sixteen metabolites of infigratinib and seven reactive intermediates (three iminium ions and four 1,4 benzoquinones) were characterized using LC-ITMS.

## Graphical abstract

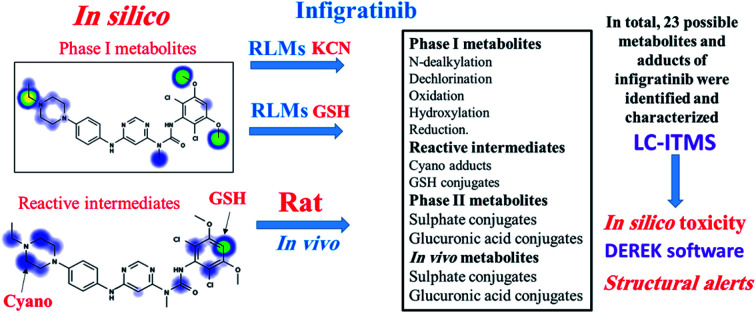

## 1. Introduction

Cholangiocarcinoma (CCA), bile duct cancer, is a type of cancer that affects the bile ducts. CCA symptoms may involve yellowish skin, abdominal pain, generalized itching, weight loss, and fever.^1^ Light-colored stool or dark urine may also occur in the majority of cases of CCA present as an unresectable disease. There is no single chemotherapy regimen that is universally used and enrollment in clinical trials is often recommended when possible.^2^ Chemotherapy agents used to treat cholangiocarcinoma include 5-fluorouracil with leucovorin,^3^ gemcitabine as a single agent,^4^ or gemcitabine plus cisplatin,^5^ irinotecan or capecitabine.^6^ Effective nontoxic treatment options for advanced CCA are needed.

Fibroblast growth factors (FGFs) and their fibroblast growth factor receptor (FGFR) pathways are crucial to cellular proliferation, cellular survival, and differentiation of many malignancies, but are especially relevant in CCA. The targeting of FGF/FGFR has become the most promising approach to treating patients with advanced/metastatic CCA.^7^ FGFRs are a family of receptor tyrosine kinase (TK) which may be upregulated in many tumor cell types and may contribute to tumor cell differentiation and proliferation, tumor angiogenesis, and tumor cell survival.^8,9^ Understanding the pathogenic mechanisms resulting from mutations, gene fusions, and gene amplifications in FGFs and FGFRs has led to therapeutic approaches for different type of cancers.

Infigratinib (INF, NVP-BGJ398) is a novel, oral small molecule used to inhibit human fibroblast growth factor receptors (FGFRs).^8^ INF is an investigational drug under development for the treatment of patients with FGFR-driven diseases, including CCA, urothelial carcinoma (bladder cancer), and achondroplasia.^10–12^ It is also under investigation for the treatment of head and neck squamous cell carcinoma,^13^ hepatocellular carcinoma (HCC),^14^ breast cancer^15^ and other type of cancers. INF is currently under phase II/III clinical trials.^16,17^ On 6 January 2020, Bridge Bio Pharma's QED Therapeutics announced that the FDA granted fast track designation to INF in adults with first-line advanced or metastatic cholangiocarcinoma and orphan drug designation for INF for cholangiocarcinoma treatment.^18^

INF's chemical name is (Z)-*N*'-(2,6-dichloro-3,5-dimethoxyphenyl)-*N*-(6-((4-(4-ethylpiperazin-1-yl) phenyl) amino) pyrimidin-4-yl)-*N*-methylcarbamimidic acid. It's adverse side effects in patients treated at the maximum tolerated dose included hyperphosphatemia (82.5%), constipation (50.9%), decreased appetite (45.6%), and stomatitis (45.6%).^13^

Identification and characterization of INF metabolites both *in vitro* and *in vivo* were performed using liquid chromatography mass spectrometry (LC-MS/MS). Metabolic activation can include the production of reactive metabolite(s) that can covalently modify proteins, which is considered the first step in developing drug-induced organ toxicities. Hence, we investigated the presence of reactive metabolites to help predict toxicities associated with INF and its drug relatives. Reactive metabolites cannot be determined *in vivo* because they can bind to endogenous materials such as DNA and proteins that prevent their detection by mass spectrometry.^19^ The chemical structure of INF contains a pyrimidine group and an *N*-ethyl piperazine ring. Compounds containing *N*-ethyl piperazine rings have been shown to undergo metabolic bioactivation, producing iminium intermediates that can be captured by a nucleophile such as potassium cyanide (KCN) to form cyano adducts.^20^ The halogenated benzene ring of the INF structure undergoes metabolic bioactivation by oxidation to form the reactive intermediate 1,4-benzoquinone that is attacked by GSH.^21^ Therefore, in the present study, we aimed to extract, identify, separate, and characterize these stabilized adducts and conjugates of INF using LC-ITMS.^22–24^ These reactive metabolites helped us predict the toxicities associated with INF.^25^

*In silico* drug metabolism is important in understanding the mechanistic basis of INF's *in vivo* biotransformation as well as changes in the properties of metabolites relative to those of the parent compound. *In silico* approaches are characterized by lower cost and shorter run time when compared to experimental steps for metabolism and toxicity profiling.^26,27^ The Xenosite CYP450 model was used for prediction of vulnerable metabolic sites in the INF chemical structure to guide the practical work. Knowing the bioactive center and structural alerts in INF's structure helped in making targeted modifications to improve its safety and retain its efficacy, confirmed using DEREK software and Xenosite reactivity model. *In silico* toxicity assessment of INF metabolites was carried out using DEREK software.

## 2. Chemicals and methods

### 2.1. Chemicals

HPLC grade solvent and analytical grade reference powders were used. Sprague Dawley rats were obtained from Prince Naif bin Abdul Aziz Health Research Center at King Saud University (KSA). Rat liver microsomes (RLMs) were prepared in-house using Sprague Dawley rats.^28,29^ INF reference powder was purchased from Med Chem Express (Princeton, NJ, USA). Formic acid (HCOOH), acetonitrile (ACN), HPLC-grade glutathione reductase (GSH) and potassium cyanide (KCN) were purchased from Sigma-Aldrich (USA). HPLC grade water was supplied by an in-house Milli-Q plus purification system (USA). Tween-80 was obtained from Eurostar Scientific Ltd (Liverpool, UK). Ethical approval for the animal experiments was obtained from the Animal Ethics Committee of King Saud University (No. KSU-SE-19-52).

### 2.2. Chromatographic conditions

Chromatographic parameters for separation of the incubation mixture are mentioned in Table 1.

**Table 1:** Adjusted parameters for the proposed LC-ITMS experiment

Liquid chromatographic parameters agilent 1200 HPLC			Mass spectrometric parameters agilent 6320 IT	
Gradient mobile phase	A: 1% HCOOH in H_2_O		Ionization source	Positive ESI
	B: CAN			Drying gas: N_2_ gas
	Flow rate: 0.250 mL min^−1^			Flow rate (10 L min^−1^)
	Run time: 85 min			Pressure (60 psi)
Agilent eclipse plus C_18_ column	Length	150 mm		ESI temperature: 350 °C
	Internal diameter	2.1 mm		Capillary voltage: 4000 V
	Particle size	3.5 μm	Collision gas	High purity N_2_
	Temperature:	23 ± 2 °C	Modes	Mass scan and MS^2^
Gradient system	Time	% B	Analyte	INF and its metabolites
	0	5	Mass parameters	Fragmentation amplitude: 1.25 V
	5	5		
	30	40		
	50	60		
	80	90		
	85	5		

### 2.3. *In silico* prediction by xenosite web predictor

Xenosite web predictor is freely available online software for the prediction of potential metabolic sites in the chemical structures of xenobiotics and other small molecules. It proposes the atomic sites of molecules changed by 9 main CYP450 isoenzymes, including 1A2, 2B6, 2A6, 2C9, 2C8, 2D6, 2C19, 3A4 and 2E1 and human liver microsomes (HLM). The software has the merit of short run time and the generated site of metabolism (SOM) score directly correlates to the possibility of metabolite generation.^30,31^ The chemical structure of INF (SMILES format) was uploaded to the website for SOM prediction.

### 2.4. *In silico* prediction of INF reactive metabolites using XenoSite reactivity model and DEREK software

XenoSite reactivity model *in silico* experiments were performed, as freely available at http://swami.wustl.edu/xenosite, to detect the vulnerable sites of reactive intermediates.^32^ This prognostic model is established on neural networks of more than 680 molecules. The software has the advantage of short run time.^32,33^ DEREK software was utilized to screen for structural alerts and to confirm our bioactivation proposal.

### 2.5. *In vitro* metabolism of INF

#### 2.5.1. RLM incubations

Rat liver microsomes (RLMs) were incubated with INF following a previously published procedure.^34,35^ Stock solution of INF in dimethyl sulfoxide (DMSO) was added to 0.08 M phosphate buffer (pH 7.4) with rat liver microsomes. The mixture was first equilibrated for 5 min in a shaking water bath at 37 °C. Incubation was initiated by adding a nicotinamide adenine dinucleotide phosphate (NADPH) solution. The final concentrations of drug, NADPH, and microsomal protein were 30 μM, 1 mM, and 1 mg mL^−1^, respectively, in a total incubation mixture volume of 1 mL. Controls were prepared either by substituting the microsomal protein or by replacing NAPDH with buffer. Samples were incubated for 90 min and the reaction was terminated with 2 mL ice-cold acetonitrile to precipitate proteins. The samples were subsequently centrifuged at 21 900*g* for 10 min. The supernatant was transferred to a fresh container and subjected to evaporation under a stream of nitrogen. The residue was reconstituted in the mobile phase and transferred to an HPLC vial for analysis. To capture reactive intermediate metabolites, the same experiment was repeated using 1.0 mM glutathione (GSH) or 1.0 mM KCN. Similarly, experiments with negative controls were performed following the above procedures. Phenytoin (2 μM) was used to assess the activity of microsomal preparation as a positive control.

#### 2.5.2. Liver hepatocytes incubation

Rat liver hepatocytes were prepared according to established procedures.^36^ Hepatocytes were incubated at 37 °C for 2 h with 30 μM INF in a rotary evaporation apparatus fitted with a custom made four-way adaptor to allow for the fitting of four 100 mL round bottom flasks. One milliliter aliquots were collected at 0, 15, 30, 60, 90 and 120 min from each flask, mixed with two volumes of acetonitrile and immediately flash frozen in liquid nitrogen. Cell viability was assessed using trypan blue at the conclusion of the incubation. Frozen samples were thawed and centrifuged at 14 000 rpm and supernatants were transferred to fresh containers and evaporated under a stream of nitrogen. The residues were reconstituted in 300 μL mobile phase and transferred to HPLC vials for analysis. Negative control experiments were performed following the above procedures.

### 2.6. *In vivo* metabolism

Six male Sprague-Dawley rats of average weight (400 g) and 1 month of age were obtained from Prince Naif bin Abdul Aziz Health Research Center at King Saud University. Each rat was housed in a special purpose metabolism cage in the animal care facility with a 12 h light/dark cycle (7:00–19:00). Rats had free access to standard water and animal food. Rats were maintained in metabolism cages for 72 h before starting the study. INF was formulated in a special solution (5% Tween 80, 30% PEG 300, 4% DMSO, HPLC H_2_O) to allow even dispersal of INF. Each rat received an INF dose depending on its weight.

The recommended INF dose is 125 mg per day until unacceptable toxicity or disease progression occurs.^11^ Average INF dose for a human is 2 mg kg^−1^. Rat doses were calculated using the following equations.^37–39^
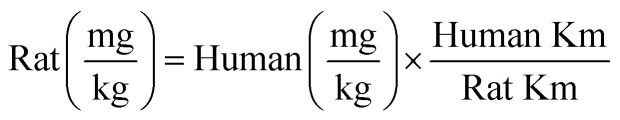

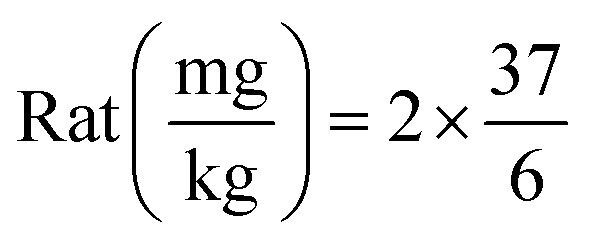

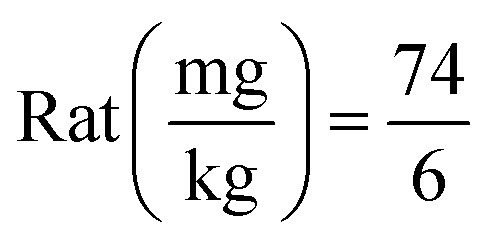

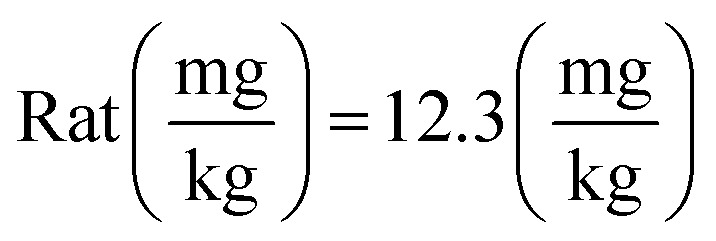


Thus, the dose used in rats was 12.3 mg kg^−1^. Oral feeding through oral gavage was used for INF dosing; rats received one dose of INF, while one rat was administered solvent alone as a negative control. Urine samples were collected from compartments fixed to the metabolism cages before INF dosing as a control sample and then at specific time points (6, 12, 18, 24, 48, 72, 96 and 120 h) following INF dosing. Urine samples were filtered through 0.45 μm syringe filters to remove particulate matter from the urine. The same volume of ACN was added to each urine sample and this mixture was vortexed for 1 min. Supernatants were removed and dried under a stream of nitrogen, then reconstituted in 0.5 mL mobile phase and transferred to HPLC Agilent vials for LC-MS-IT-MS analysis. Control urine samples obtained from rats before drug dosing were prepared similarly and analyzed by LC-IT-MS to obtain control chromatograms.

## 3. Results and discussion

### 3.1. *In silico* prediction of INF metabolism and reactivity

**Fig. 1:**
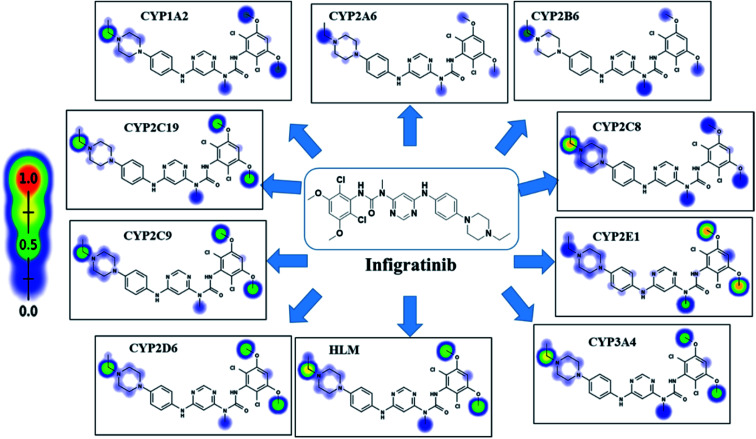
Predicted atomic sites of metabolism for INF by Xenosite web predictor.

INF SOMs were proposed using 9 key CYP450 isoenzymes including 1A2, 2A6, 2B6, 2C8, 2C9, 2C19, 2D6, 2E1 and 3A4 and HLM. The results of *in silico* SOM predictions for INF are shown in Fig. 1. The outcomes were obtained in the form of visual image in terms of the color gradient intensity, including color scale bar. The highest probability of metabolism at atomic site was denoted with red color (highest score: 1) while a white color implies zero SOM score indicating no possibility for metabolism at atomic site. The suggested atomic sites for the metabolism in INF structure are the two methoxy groups, the ethyl group attached to the piperazine ring, the methyl group attached to the *N*-pyrimidine ring and the α carbon atoms attached to the nitrogens of the piperazine ring. These results matched practical experiments, in which from M1 to M7 and M18 to M21 matched *in silico* predictions.

**Fig. 2:**
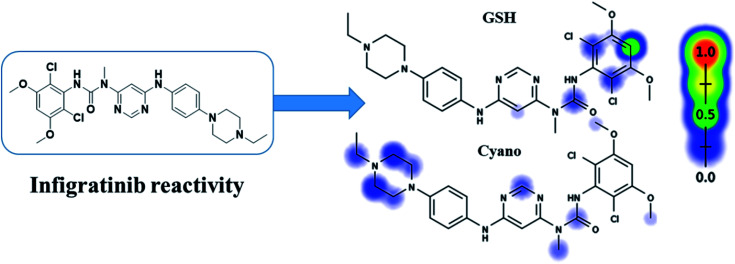
Predicted bioactive sites of INF by Xenosite web predictor, including GSH and cyano bioactive centers.

INF reactivity was predicted using cyano and GSH models in Xenosite as shown in Fig. 2. Consecutively, depending on *in silico* postulations and literature knowledge, a list of proposed metabolites and reactive intermediates was prepared and utilized as a guide during practical work. The cyano reactivity model indicates the highest probability of *N*-ethyl piperazine ring bioactivation. The GSH reactivity model indicates the highest probability of 2,6-dichloro-3,5-dimethoxyphenyl ring bioactivation, indicated by the dark color. Results from *in silico* reactivity models match the practical results. Other places of bioactivation have low priorities as indicated by the faint color.

### 3.2. Fragmentation analysis of INF

**Fig. 3:**
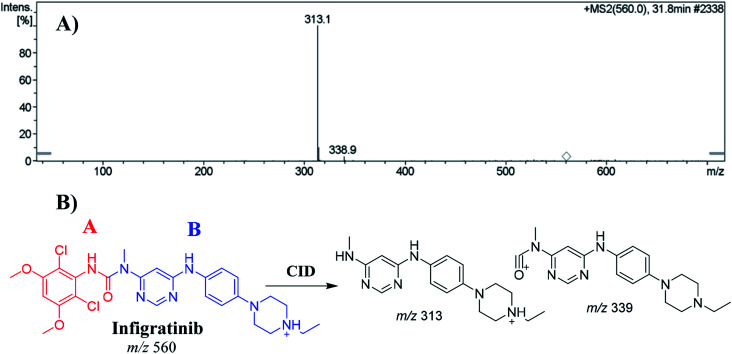
Positive ion MS^2^ of INF at 31.8 min (A). Fragment ions of INF (B).

The INF parent ion peak (PIP) appeared at 31.8 min in the total ion chromatogram (TIC). Fragmentation of PI at *m*/*z* 560 resulted in two daughter ions at *m*/*z* 339 and at *m*/*z* 313 (Fig. 3A). The chemical structure of INF was marked into two substructures as A (red color) and B (blue color) to facilitate localization of metabolic reactions (Fig. 3B).

### 3.3. Identification of INF *in vitro* phase I and phase II metabolites

Purified extracts recovered from RLM assays were subjected to LC-IT-MS. Seven phase I metabolic reactions were generated by α hydroxylation, *N*-demethylation, *N*-dealkylation, *O*-demethylation and dechlorination (Table 2). Two sulphate conjugates and one glucuronic acid conjugate were identified when INF was incubated with isolated perfused rat liver hepatocytes (Table 3).

**Table 2:** Phase I metabolites of INF identified in MS and MS^2^ scans

	MS scan	Major daughter ions	*t*_R_ (min)	Proposed metabolic reaction
INF	560	339, 313	31.8	INF + H
M1	576	559, 329, 312, 205	33.7	α Hydroxylation at piperazine ring
M2	546	528, 339, 313	31.7	*O*-Demethylation
M3	532	311, 285	35.1	*N*-Dealkylation
M4	510	492, 325	24.6	Dechlorination of two chlorine groups then hydroxylation and *N*-demethylation
M5	528	510, 313	20.2	Dechlorination of one chlorine group then hydroxylation and *O*-dealkylation
M6	542	340, 313	20.7	Dechlorination of one chlorine group then hydroxylation
M7	524	506, 312	29.1	Dechlorination of two chlorine groups then hydroxylation

**Table 3:** *In vitro* phase II INF metabolites

	MS scan	Most abundant fragment ions	*R*_t_ (min)	Metabolic reaction
**Sulphate conjugates**				
M8	626	546	29.5	*O*-Demethylation and conjugation of sulphate
M9	608	591	26.5	*N*-Demethylation, dechlorination then hydroxylation and conjugation of sulphate group

**Glucuronide conjugates**				
M10	722	546, 313	24.3	*O*-Demethylation and conjugation of glucuronic acid

All *in vitro* phase I and phase II metabolites fragmentation patterns and structural details are listed in the ESI† (Fig. S1–S10).

### 3.4. Identification of *in vivo* metabolites of INF

PI mass spectra comparison between control urine samples and urine extracts, as well as PI comparison of INF and its proposed metabolites (Table 4), permitted the identification of four *in vivo* phase I and three phase II metabolites. Metabolic reactions for *in vivo* phase I metabolites were proposed to be *N*-dealkylation, *N*-demethylation, *O*-demethylation and hydroxylation, while for phase II metabolites, the results suggested two sulphate and one glucuronic acid conjugation. M17, M18, M22 and M23 metabolites were previously detected in *in vitro* INF phase I and phase II metabolism.

**Table 4:** Reactive metabolites of INF

	MS scan	Most abundant fragment ions	*R*_t_ (min)	Metabolic reaction
**Cyano adducts**				
M11	585	558, 338	39.4	Attack of KCN at bioactivated *N*-ethyl piperazine ring
M12	571	544, 350, 324	50.7	*N*-Demethylation of *N*-methylformamide group and attack of KCN at bioactivated *N*-ethyl piperazine ring
M13	599	572, 378, 352	56.8	α-Carbonyl formation and attack of KCN at bioactivated *N*-ethyl piperazine ring

**GSH conjugates**				
M14	881	822, 574	55.4	*O*-Demethylation, dechlorination followed by hydroxylation, triple hydroxylation at piperazine moiety of INF structure and then conjugation of GSH at 1,4-benzoquinone
M15	793	720 486	51.9	*N*-Dealkylation, reduction, *O*-demethylation; dechlorination followed by hydroxylation and conjugation of GSH at the bioactivated 1,4-benzoquinone
M16	837	748, 708	53.9	Hydroxylation, reduction, *O*-demethylation, and dechlorination, followed by hydroxylation, and finally, conjugation of the GSH at the bioactivated 1,4-benzoquinone
M17	805	676, 308	52.7	*O*-Demethylation, *N*-dealkylation, dechlorination followed by hydroxylation and conjugation of GSH at bioactivated 1,4-benzoquinone

All *in vivo* phase I and phase II metabolites' fragmentation patterns and structural details are listed in the ESI† (Fig. S11–S13).

### 3.5. Identification of *in vitro* INF reactive metabolites

The same metabolic reaction between INF and RLMs was performed in the presence of 1.0 mM KCN and 1.0 mM GSH to trap iminium and 1,4-benzoquinone intermediates, respectively. Three cyanide adducts and four GSH conjugates were identified (Table 5), indicating that the *N*-ethyl piperazine ring and benzene ring at part A of the INF structure could be bioactivated and then trapped by the nucleophile cyanide ion and GSH, respectively.^20,28,40,41^

**Table 5:** *In vivo* phase I and phase II INF metabolites

	MS scan	Most abundant fragment ions	*R*_t_(min)	Metabolic reaction
**Phase I metabolites**				
M1	576	559, 329, 312	32.5	Hydroxylation at *N*-ethyl piperazine ring
M3	532	311, 285	30.2	*N*-Dealkylation of *N*-ethyl piperazine ring
M20	592	575, 345, 328, 298, 221	30.8	Double hydroxylation at *N*-ethyl piperazine ring
M21	490	472, 314, 163	27.4	*N*-Dealkylation, *N*-demethylation, *O*-demethylation of two methyl groups

**Phase II metabolites**				
M22	752	576, 505, 291	29.5	*N*-Glucuronic acid conjugate at *N*-ethyl piperazine ring
M8	626	546, 312	26.3	*O*-Demethylation and *O*-sulphate conjugation
M9	608	528, 415, 342	33.2	*N*-Demethylation, dechlorination then hydroxylation and conjugation of sulphate group

#### 3.5.1. Identification of INF cyano adducts

##### 3.5.1.1. Identification of the M11 cyano adduct reactive metabolite of INF

**Fig. 4:**
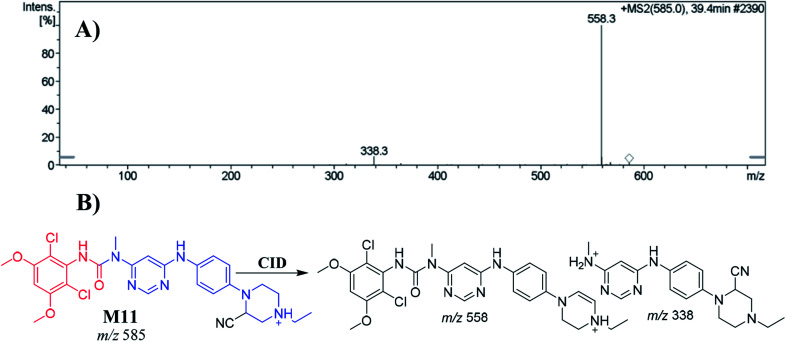
Positive ion MS^2^ mass spectrum of M11 at 39.4 min (A). Proposed structural formulas of M11 and its corresponding MS^2^ fragments (B).

The M11 PIP appeared at 39.4 min. The metabolic reaction that occurred in M11 potentially included a cyano nucleophile attack at the bioactivated piperazine ring, forming a cyano adduct. The collision-induced dissociation (CID) of M7 at *m*/*z* 585 gave daughter ions at *m*/*z* 558 and *m*/*z* 338 (Fig. 4A). The PI at *m*/*z* 558 suggested a loss of hydrogen cyanide molecules (27 Da) that was consistent with cyano addition. Compared to PIs of INF, the product ion at *m*/*z* 338 confirmed the location of the cyanide ion addition to the *N*-ethyl piperazine ring (Fig. 4B).

##### 3.5.1.2. Identification of the M12 cyano adduct reactive metabolite of INF

**Fig. 5:**
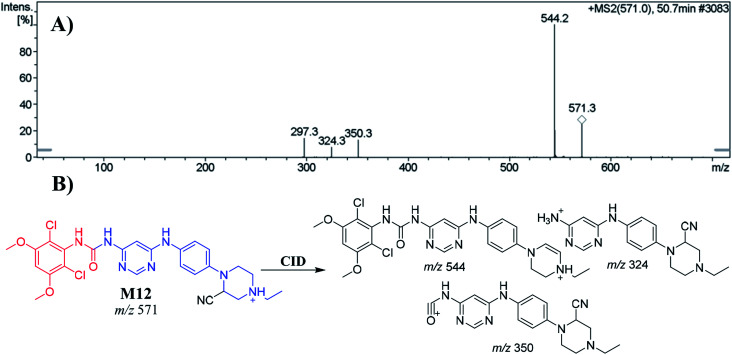
Positive ion MS^2^ mass spectrum of M12 at 50.7 min (A). Proposed structural formulas of M12 and its corresponding MS^2^ fragments (B).

The M12 PIP appeared at 50.7 min. The metabolic reactions that occurred in M12 were *N*-demethylation of the nitrogen attached to the pyrimidine ring and cyano nucleophile attack at the bioactivated piperazine ring, forming the cyano adduct. CID of the M12 ion at *m*/*z* 571 produced three characteristic product ions at *m*/*z* 544, *m*/*z* 350 and *m*/*z* 324 (Fig. 5A). The PI at *m*/*z* 544 suggested a loss of hydrogen cyanide molecules (27 Da) consistent with the cyano addition. Compared to the PIs of INF, the ions produced at *m*/*z* 324 and *m*/*z* 350 confirmed that the location of the cyanide ion addition was at the *N*-ethyl piperazine ring (Fig. 5B).

##### 3.5.1.3. Identification of the M13 cyano adduct reactive metabolite of INF

**Fig. 6:**
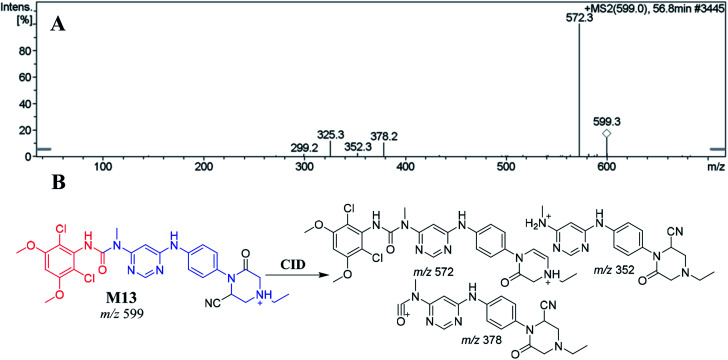
Positive ion MS^2^ mass spectrum of M13 at 56.8 min (A). Proposed structural formulas of M13 and its corresponding MS^2^ fragments (B).

M13 appeared at 56.80 min. The metabolic reactions that occurred in M13 suggested the formation of an α-carbonyl on the *N*-ethyl piperazine ring group and a cyano nucleophile attack on the bioactivated piperazine ring to form the cyano adduct. CID of the M13 ion at *m*/*z* 599 produced three characteristic product ions at *m*/*z* 572, *m*/*z* 378 and *m*/*z* 352 (Fig. 6A). The PI at *m*/*z* 572 indicated a loss of hydrogen cyanide molecules (27 Da) that confirmed cyano addition. Compared to the PIs of INF, the product ions at *m*/*z* 378 and *m*/*z* 352 confirmed cyanide ion addition on the *N*-ethyl piperazine ring (Fig. 6B).

#### 3.5.2. Identification of INF GSH conjugates

##### 3.5.2.1. Identification of the M14 GSH conjugate of INF

**Fig. 7:**
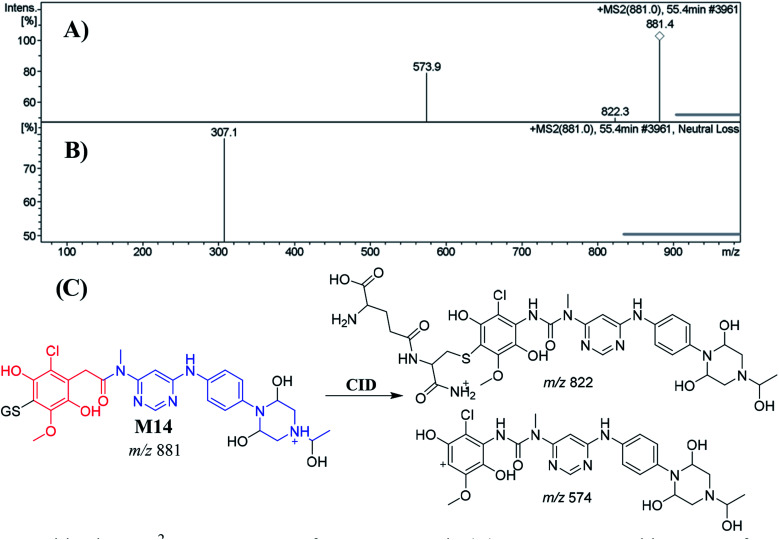
Positive ion MS^2^ mass spectrum of M14 at 55.4 min (A). Constant neutral loss scan of M14 (B). Proposed structural formulas of M14 and the corresponding MS^2^ fragments (C).

M14 appeared at 55.4 min. The metabolic pathways that occurred in M14 suggested *O*-demethylation and dechlorination followed by hydroxylation, triple hydroxylation at piperazine moiety of INF structure and then conjugation of GSH at 1,4-benzoquinone. M14 formation indicated 1,4-benzoquinone generation in the *in vitro* metabolism of INF. CID of the M14 ion at *m*/*z* 881 produced two characteristic fragment ions at *m*/*z* 822 and *m*/*z* 574 (Fig. 7A). The product ion at *m*/*z* 574 resulted from the cleavage of the GSH moiety and confirmed GSH conjugate formation. The product ion at *m*/*z* 822 suggested a loss of acetic acid molecules from the GSH conjugate (Fig. 7C). An LC-MS/MS screening for the GSH conjugate was performed by constant neutral loss scan monitoring of ions that lost 307 Da (Fig. 7B).^42^

##### 3.5.2.2. Identification of the M15 GSH conjugate of INF

**Fig. 8:**
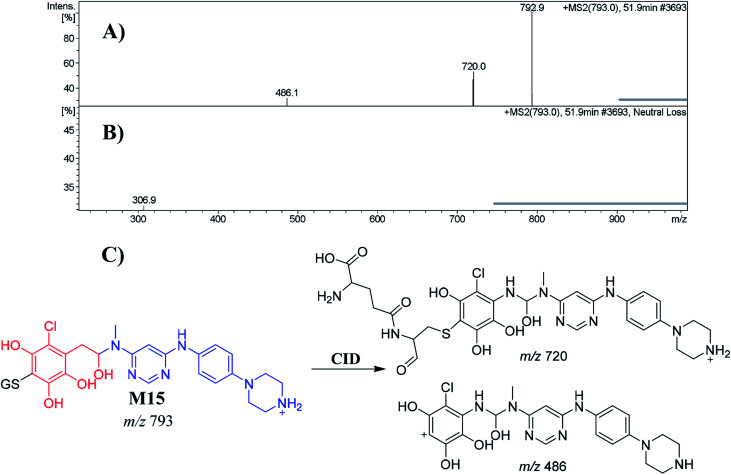
Positive ion MS^2^ mass spectrum of M15 at 51.9 min (A). Constant neutral loss scan of M15 (B). Proposed structural formulas of M15 and its corresponding MS^2^ fragments (C).

M15 appeared at 51.9 min. The metabolic pathways that occurred in M15 were *N*-dealkylation, reduction, *O*-demethylation, dechlorination followed by hydroxylation and conjugation of GSH at the bioactivated 1,4-benzoquinone. M15 formation indicated 1,4-benzoquinone generation in the *in vitro* metabolism of INF. The CID of the M15 ion at *m*/*z* 793 produced two characteristic fragment ions at *m*/*z* 720 and *m*/*z* 486 (Fig. 8A). The product ion at *m*/*z* 486 resulted from the cleavage of the GSH moiety, consistent with GSH conjugate formation. The product ion at *m*/*z* 720 suggested a loss of acetic acid molecules from the GSH conjugate (Fig. 8C). Another LC-MS/MS screening for a GSH conjugate was performed by constant neutral loss scan monitoring of ions that lose 307 Da (Fig. 8B).^42^

##### 3.5.2.3. Identification of the M16 GSH conjugate of INF

**Fig. 9:**
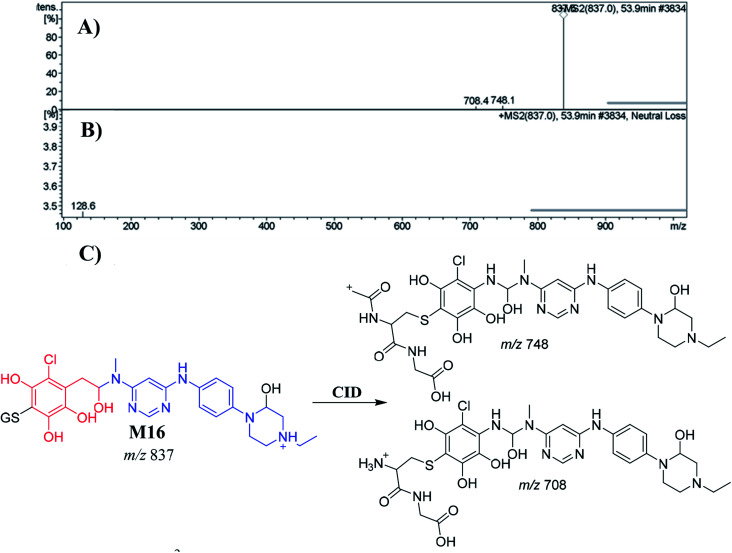
Positive ion MS^2^ mass spectrum of M16 at 53.9 min (A). Constant neutral loss scan of M16 (B). Proposed structural formulas of M16 and corresponding MS^2^ fragments (C).

M16 appeared at 53.9 min. The metabolic pathways that occurred in M16 were hydroxylation, reduction, *O*-demethylation, and dechlorination, followed by hydroxylation, and finally conjugation of the GSH at the bioactivated 1,4-benzoquinone. M16 formation indicated that 1,4-benzoquinone generation occurred in the *in vitro* metabolism of INF. The CID of the M16 ion at *m*/*z* 837 produced two characteristic fragment ions at *m*/*z* 748 and *m*/*z* 708 (Fig. 9A). The product ion at *m*/*z* 708 resulted from the cleavage of the pyroglutamic acid moiety (129 Da), consistent with GSH conjugate formation.^43^ The product ion at *m*/*z* 748 indicated a loss of amino butanoic acid molecules from the GSH conjugate (Fig. 9C). An LC-MS/MS screening for GSH conjugates was performed by constant neutral loss scan monitoring of ions that lost 129 Da (Fig. 9B).^43^

##### 3.5.2.4. Identification of the M17 GSH conjugate of INF

**Fig. 10:**
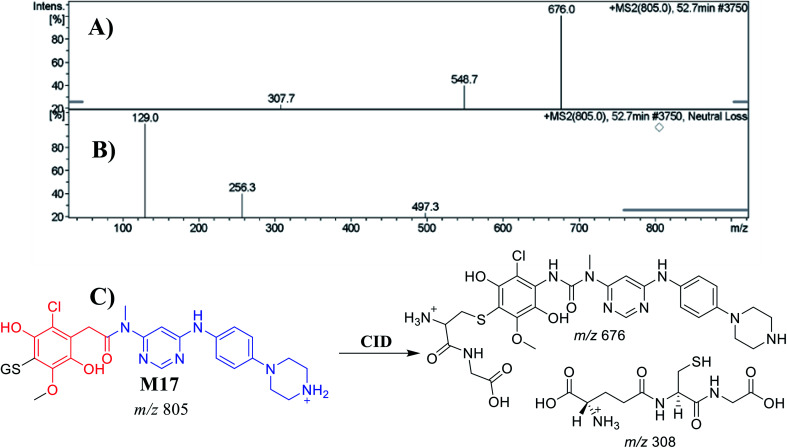
Positive ion MS^2^ mass spectrum of M17 at 52.7 min (A). Constant neutral loss scan of M17 (B). Proposed structural formulas of M17 and corresponding MS^2^ fragments (C).

M17 appeared at 52.7 min. The metabolic pathways that occurred in M17 were *O*-methylation, *N*-dealkylation and dechlorination, followed by hydroxylation and conjugation of the GSH at the bioactivated 1,4-benzoquinone. M17 formation indicated that 1,4-benzoquinone generation occurred in the *in vitro* metabolism of INF. The CID of the M18 ion at *m*/*z* 805 produced two characteristic fragment ions at *m*/*z* 676 and *m*/*z* 308 (Fig. 10A). The product ion at *m*/*z* 676 resulted from the cleavage of the pyroglutamic acid moiety (129 Da), consistent with GSH conjugate formation.^43^ The product ion at *m*/*z* 308 indicated a loss of GSH conjugate (Fig. 10C). An LC-MS/MS screening for GSH conjugates was performed by constant neutral loss scan monitoring of ions that lost 129 Da (Fig. 10B).^43^

### 3.6. Proposed bioactivation pathways of INF

**Fig. 11:**
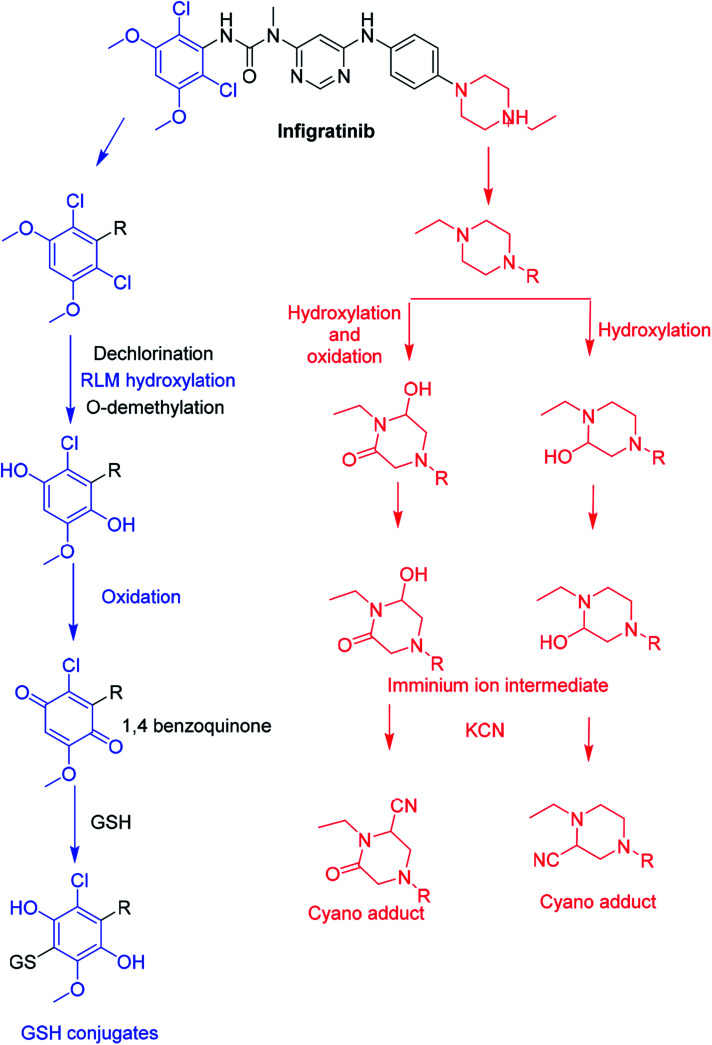
Proposed pathways for INF bioactivation.

The bioactivation pathways of INF are proposed in Fig. 11. M11, M12 and M13 cyanide adducts indicated metabolic generation of iminium intermediates at the *N*-ethyl piperazine ring during INF metabolism. The bioactivation mechanism was proposed to be hydroxylation of the *N*-ethyl piperazine ring in INF followed by dehydration that resulted in intermediate generation of iminium ion, which is a very reactive and unstable nucleophile. These reactive intermediates were captured using KCN to form stable cyanide adducts that could be detected with LC-IT-MS. The proposed bioactive mechanism of the iminium intermediate has been previously reported using drugs containing cyclic tertiary amine rings.^20,34,44^ The 1,4-benzoquinone intermediate formation in INF metabolism was confirmed using GSH as a capturing agent. We hypothesized that the bioactivation mechanism included *O*-demethylation and dechlorination followed by hydroxylation then oxidation to form 1,4-benzoquinone that could be attacked by the GSH forming conjugate (*e.g.* M14, M15, M16 and M17; Fig. 11).^40,41^

### 3.7. *In silico* prediction of INF metabolites using DEREK software

**Fig. 12:**
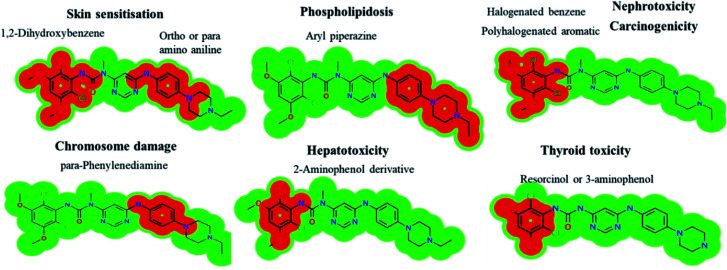
DEREK outcomes showing structural alerts with the proposed side effects of INF. Red color indicates the structural alerts.

INF metabolites contained six structural alerts after DEREK software was utilized to screen for structural alerts and to confirm our bioactivation proposal. All side effects are related to two main substructures (halogenated aromatic and aryl piperazine) that were bioactivated. INF and its metabolites show seven side effects due to the presence of structural alerts as proposed by DEREK software (Fig. 12). All metabolites show skin sensitization and chromosome damage due to the presence of 1,2-dihydroxybenzene and phenylenediamine substructures, respectively. M21 shows thyroid toxicity due to the presence of a resorcinol substructure.

The DEREK results for INF and its metabolites are summarized in Table 6.

**Table 6:** *In silico* toxicity results of INF using DEREK software

INF and its metabolites	Nephrotoxicity	Phospholipidosis	Hepatotoxicity	Carcinogenicity	Skin sensitization	Chromosome damage	Thyroid toxicity
Structural alert	Halogenated benzene	Aryl piperazine or analogue	2-Aminophenol derivative	Polyhalogenated aromatic	*ortho* or *para* Amino- or hydroxy-aniline	*para*-Phenylenediamine	
INF	Equivocal	Plausible	NA	Plausible	Plausible	Plausible	NA
M1	Equivocal	NA	NA	Plausible	Plausible	Plausible	NA
M2	Equivocal	Plausible	NA	Plausible	Plausible	Plausible	NA
M3	Equivocal	Plausible	NA	Plausible	Plausible	Plausible	NA
M4	NA	Plausible	Equivocal	Plausible	Plausible	Plausible	NA
M5	Equivocal	Plausible	Equivocal	NA	Plausible	Plausible	NA
M6	Equivocal	Plausible	Equivocal	Plausible	Plausible	Plausible	NA
M7	NA	Plausible	Equivocal	Plausible	Plausible	Plausible	NA
M8	NA	Plausible	NA	Plausible	Plausible	Plausible	NA
M9	NA	Plausible	NA	NA	Plausible	Plausible	NA
M10	NA	Plausible	NA	NA	Plausible	Plausible	NA
M20	Equivocal	NA	NA	Plausible	Plausible	Plausible	NA
M21	Equivocal	Plausible	NA	Plausible	Plausible	Plausible	Plausible
M22	Equivocal	NA	NA	NA	Plausible	Plausible	NA

## 4. Conclusions

**Fig. 13:**
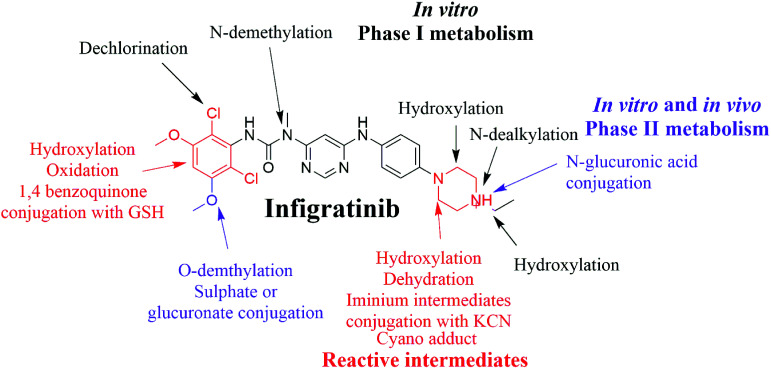
Chemical structure of INF and its bioactivation pathways that include iminium and an electro-deficient conjugated system.

In summary, an *in silico* tool has predicted the three most vulnerable SOMs in the structure of INF. The suggested atomic sites for metabolism in the INF structure were the same as seen in the generated metabolites in phase I metabolism. Additionally, the same groups were bioactivated as predicted by *in silico* experiments. The *in silico* tool has predicted the three most vulnerable SOMs in the structure of INF for phase I metabolites and also the suspected bioactive centers. These probabilities were used as a guide during practical work. Seven *in vitro* phase I metabolites were identified for INF. *N*-demethylation, *N*-dealkylation, *O*-demethylation, dechlorination, and hydroxylation were the metabolic pathways involved in INF metabolism. Seven potential reactive metabolites, including three iminium ions and four 1,4-benzoquinone intermediates, were also identified and the mechanisms of their generation were proposed (Fig. 13). The generation of these reactive intermediates during INF metabolism improves our understanding of the reasons behind the toxic side effects of INF. Three *in vitro* phase II metabolites of INF were also identified: two sulphate conjugates and one glucuronic acid conjugate. Four *in vivo* phase I and three *in vivo* phase II metabolites were identified for INF and included metabolic pathways for *in vivo* phase I *N*-demethylation, *N*-dealkylation, *O*-demethylation and hydroxylation, while the metabolic pathways for *in vivo* phase II metabolites produced sulphate and glucuronic acid conjugates (Table 7). An *in silico* toxicological report for INF and its metabolites was proposed utilizing DEREK software. These complete data for *in vivo* and *in vitro* metabolic profiling in addition to different *in silico* experiments could be utilized for designing new compounds with improved pharmacological properties and reduced side effects.

**Table 7:** List of INF metabolites

Metabolites	RLM	Hepatocytes	GSH	KCN	*In silico*	*In vivo* phase II	Metabolites	RLM	Hepatocytes	GSH	KCN	*In vivo* phase I	*In vivo* phase II
M1	✓				✓		M13			✓			
M2	✓				✓		M14				✓		
M3	✓				✓		M15				✓		
M4	✓				✓		M16				✓		
M5	✓				✓		M17				✓		
M6	✓				✓		M8		✓				
M7	✓				✓		M9		✓				
M18					✓		M10		✓				
M19					✓		M11			✓			
M20					✓		M22						✓
M21					✓		M23						✓
M12			✓				M24						✓

## Abbreviations

LC-ITMS: Liquid chromatography ion trap mass spectrometry
INF: Infigratinib
ACN: Acetonitrile
ESI: Electrospray ionization
RLMs: Rat liver microsomes
TKIs: Tyrosine kinase inhibitors
PIP: Parent ion peak
TIC: Total ion chromatogram
CID: Collision-induced dissociation

## Conflicts of interest

The authors declare no conflicts of interest.

## Supplementary Material

RA-010-C9RA10871H-s001
