# Supplementary material for: Identification and characterization of in silico, in vivo, in vitro, and reactive metabolites of infigratinib using LC-ITMS: bioactivation pathway elucidation and in silico toxicity studies of its metabolites
Source: RSC Adv. 2020 Apr 23;10(28):16231–44. doi: 10.1039/c9ra10871h (PMC9052791; doi:10.1039/c9ra10871h)
Supplement: RA-010-C9RA10871H-s001 [file RA-010-C9RA10871H-s001.pdf]

Electronic Supplementary Material (ESI) for RSC Advances.
This journal is © The Royal Society of Chemistry 2020

Supplementary data

1. *In vitro* and *in vivo* drug metabolism of INF

1.1. Identification of infigratinib *in vitro* phase I metabolites

1.1.1. Identification of the INF M1 metabolites

The M1 PIP at 33.7 min. which represented hydroxylation metabolite of INF. Collision induced dissociation (CID) of the molecular ion peak (MIP) at *m/z* 576 generated fragment ions at *m/z* 559, *m/z* 329, *m/z* 312 and *m/z* 205 (Fig. S1A). The production of a daughter ion at *m/z* 205 suggested that the hydroxylation occurred at piperazine moiety (part B of INF), which matched with the other fragment ions at *m/z* 559, *m/z* 329 and *m/z* 312(Fig. S1B).

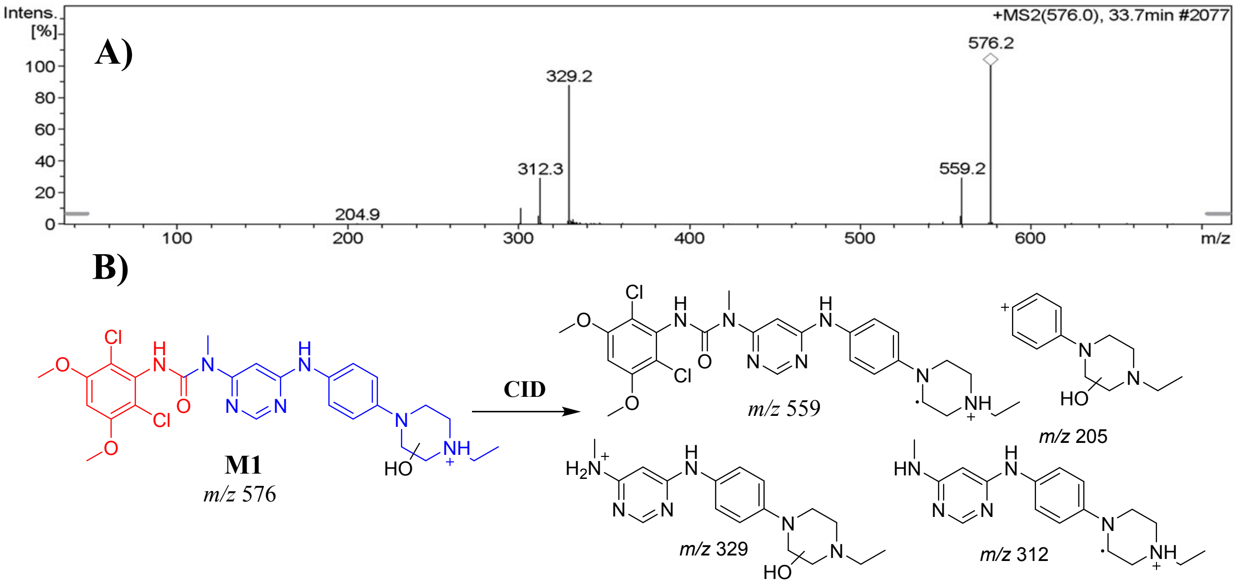

Fig. S1. Positive ion MS² mass spectrum of M1 at 33.7 min (A). Proposed structural formulas of M1 and corresponding MS² fragments (B).

1.1.2. Identification of the INF M2 metabolite

The M2 PIP appeared at 31.7 min. which represented an *O*-demethylation metabolite of INF. CID of the MIP at *m/z* 546 generated fragment ions at *m/z* 339 and *m/z* 313 (Fig. S2A). The production of a daughter ion at *m/z* 313 suggested that no metabolic change occurred at part B of INF, which matched with the other fragment ions at *m/z* 339 (Fig. S2B).

Fig. 6

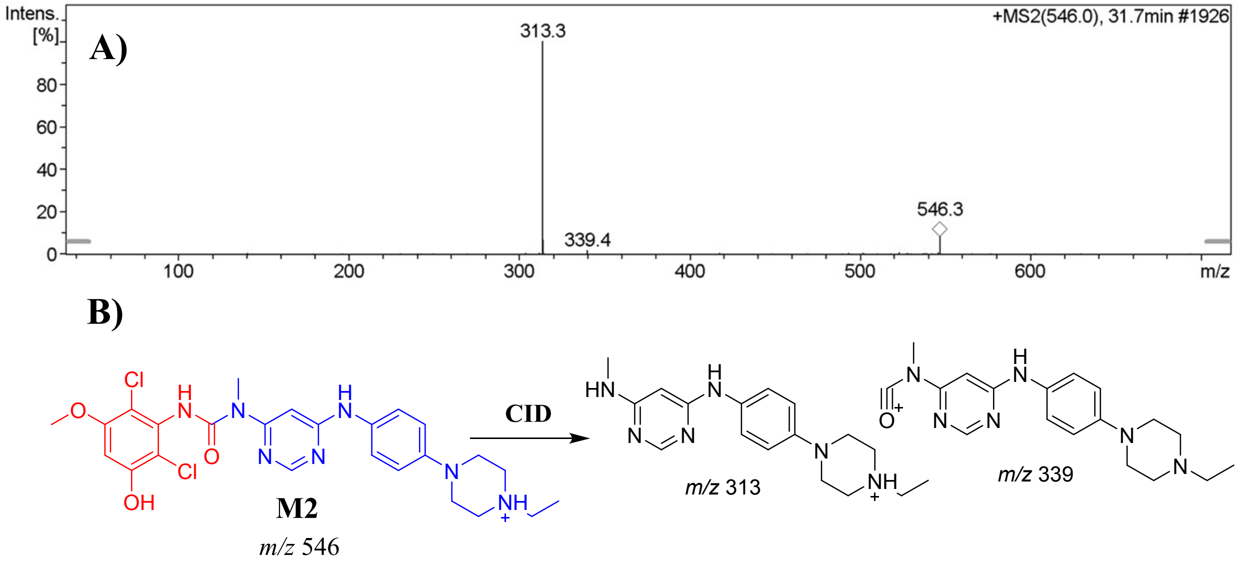

Fig. S2. Positive ion MS^2 mass spectrum of M2 at 31.7 min (A). Proposed structural formulas of M2 and corresponding MS^2 fragments (B).

1.1.3. Identification of the INF M3 metabolite

The M3 PIP appeared at 35.1 min. which represented *N*-dealkylation metabolite of INF. CID of the MIP at *m/z* 532 generated fragment ions at *m/z* 311 and *m/z* 285 (Fig. S3A). The production of a daughter ion at *m/z* 285 suggested that the *N*-dealkylation occurred at piperazine moiety (part B of INF), which matched with the other fragment ions at *m/z* 339 (Fig. S3B).

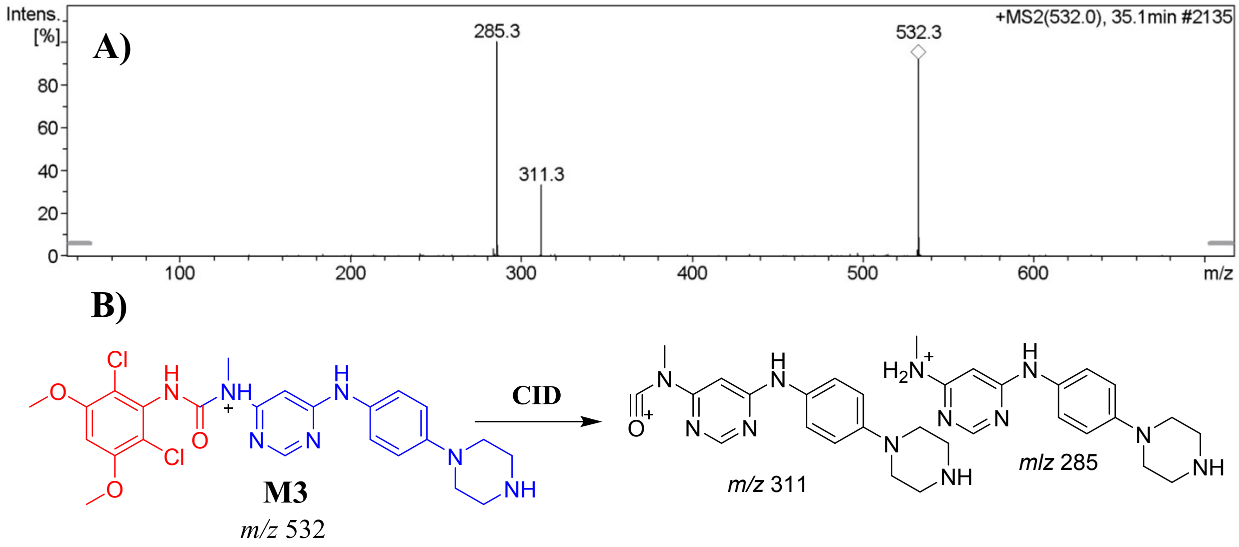

Fig. S3. Positive ion MS^2 mass spectrum of the M3 peak (A). Proposed structural formulas of M3 and corresponding MS^2 fragments (B).

1.1.4. Identification of the INF M4 metabolite

The M4 PIP appeared at 24.6 min. The M4 metabolite was the net product of *N*-demethylation and dechlorination of two chlorine groups followed by hydroxylation at the same sites. CID of MIP at *m/z* 510 generated fragment ions at *m/z* 492 and *m/z* 325 (Fig. S4A). The production of a daughter ion at *m/z* 325 suggested that an *N*-demethylation reaction occurred at part B of the INF structure while dechoronation and hydroxylation occurred at part A of INF structure, which matched with the other fragment ions at *m/z* 492 (Fig. S4B).

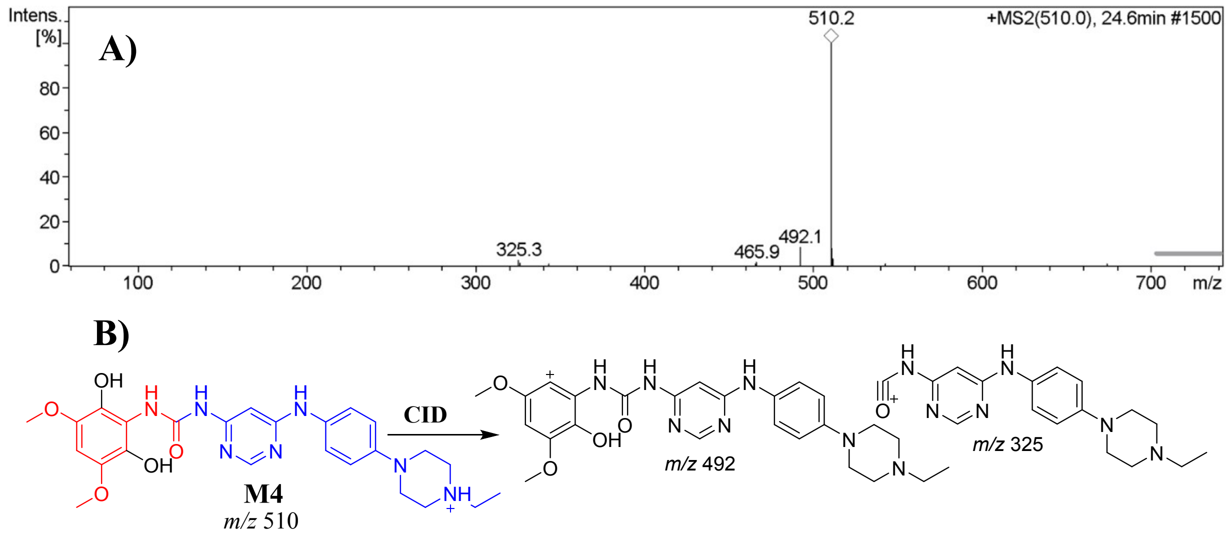

Fig. S4. Positive ion MS^2 mass spectrum of M4 at 24.6 min (A). Proposed structural formulas of M4 and corresponding MS^2 fragments (B).

1.1.5. Identification of the INF M5 metabolite

The M5 PIP appeared at 20.2 min. The M5 metabolite was the net product of *O*-demethylation and dechlorination of one chlorine groups followed by hydroxylation at the same sites. CID of MIP at *m/z* 528 generated fragment ions at *m/z* 510 and *m/z* 313 (Fig. S5A). The production of a daughter ion at *m/z* 510 suggested the loss of a hydroxyl group from part A of the INF structure, while the daughter ion at *m/z* 313 suggested that there was no change in part B of INF (Fig. S5B).

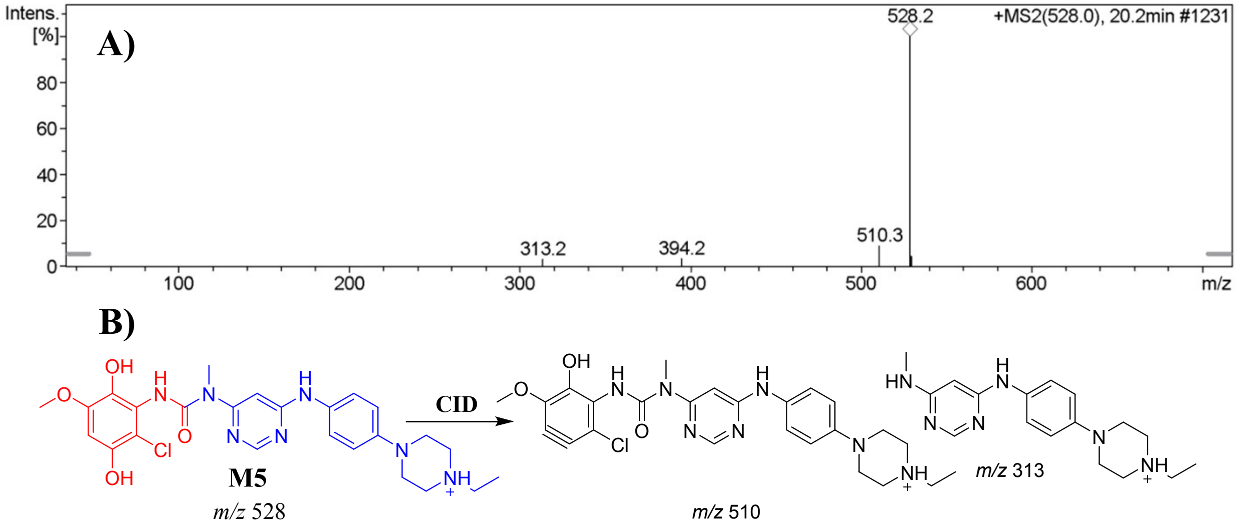

Fig. S5. Positive ion MS^2 mass spectrum of M5 at 20.2 min (A). Proposed structural formulas of M5 and corresponding MS^2 fragments (B).

1.1.6. Identification of the M6 INF metabolite

The M6 PIP appeared at 20.7 min. The metabolite M6 was the net product of dechlorination of one chlorine group followed by hydroxylation at the same site. The CID of the MIP at *m/z* 542 generated fragment ions at *m/z* 340 and *m/z* 313 (Fig. S6A). The production of the daughter ion at *m/z* 313 indicated that no metabolic change occurred at part B of INF, which was consistent with the other fragment ions at *m/z* 340 (Fig. S6B).

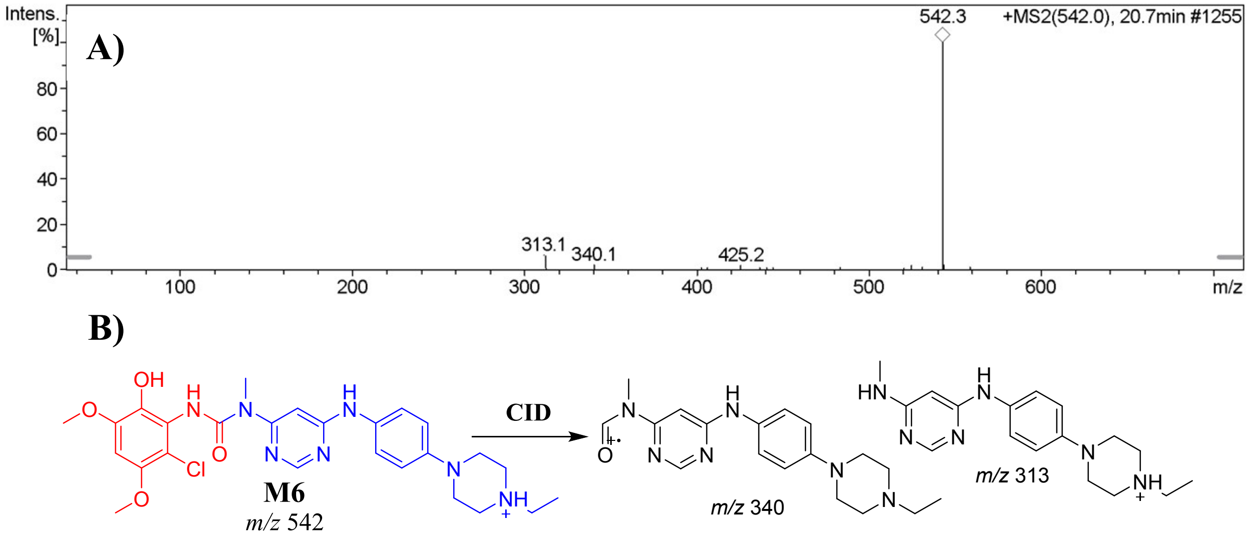

Fig. S6. Positive ion MS^2 mass spectrum of M6 at 20.7 min. (A). Proposed structural formulas of M6 and its corresponding MS^2 fragments (B).

1.1.7. Identification of the INF M7 metabolite

The M7 PIP appeared at 29.1 min. The M7 metabolite was the net product of dechlorination of two chlorine groups followed by hydroxylation at the same sites. The CID of M7 at *m/z* 524 gave daughter ions at *m/z* 506 and *m/z* 312. The production of the daughter ion at *m/z* 506 indicated the loss of a hydroxyl group from the benzene ring (part A) (Fig. S7A), while the daughter ion at *m/z* 312 suggested that there was no change at part B in the INF structure (Fig. S7B).

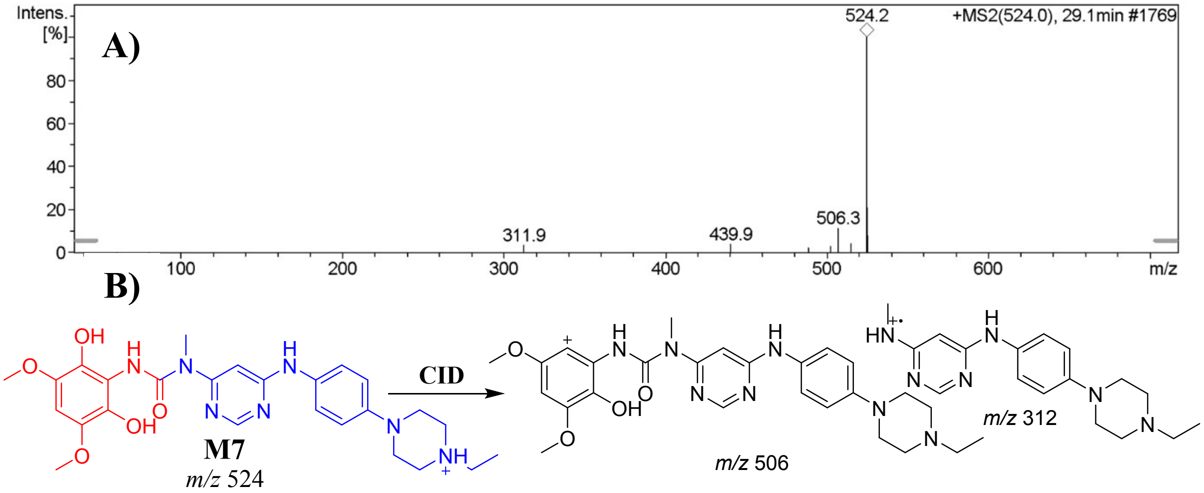

Fig. S7. Positive ion MS^2 mass spectrum of M7 at 29.1 min. (A). Proposed structural formulas of M7 and corresponding MS^2 fragments (B).

1.2. Identification of INF *in vitro* phase II metabolites

Two sulphate conjugates and one glucuronic acid conjugate were identified when INF was incubated with isolated perfused rat liver hepatocytes.

1.2.1. Identification of the INF M8 metabolite

The M8 appeared at 26.6 min. and had a MIP at *m/z 626*, which was 80 Da higher than that of its precursor ion (M2) at *m/z* 546 that was proposed to be a sulfate conjugation metabolite of INF[38]. CID of MIP at *m/z* 626 generated fragment ion at *m/z* 546 (Fig. S8A). which was 80 Da less than the precursor ion and suggested a loss of a sulphate group from the benzene ring in part A of the INF structure (Fig. S8C). Another LC-MS/MS screening for sulfate adducts was performed by constant neutral loss scan monitoring of ions that lose 80 Da[39] (Fig. S8B).

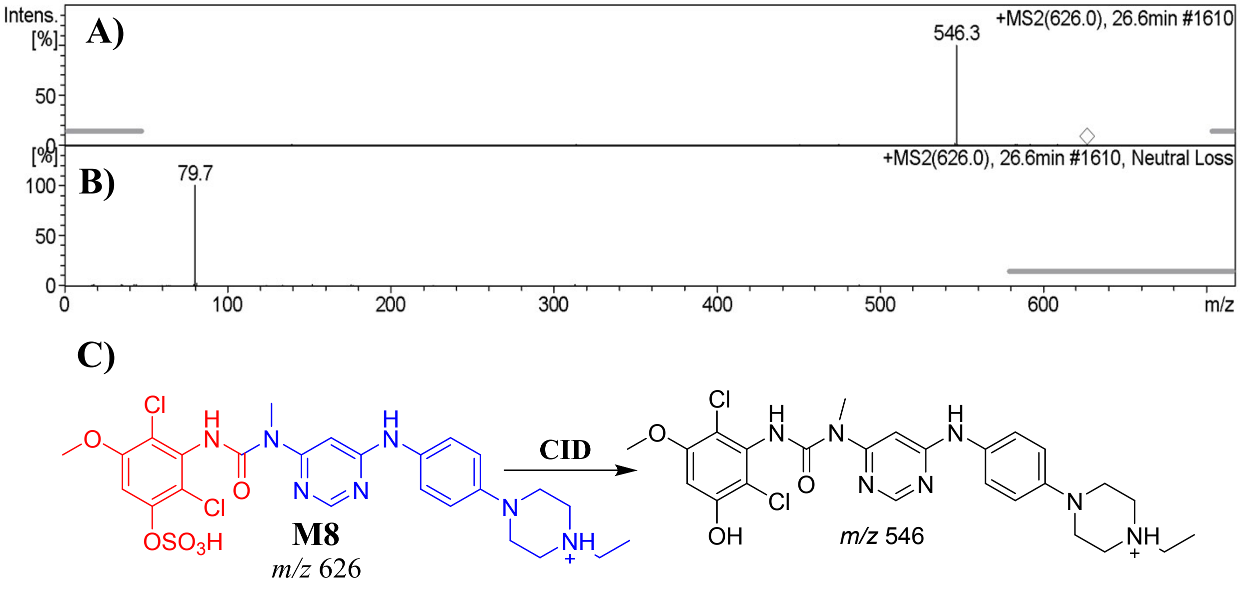

Fig. S8. Positive ion MS² mass spectrum of M8 at 26.6 min (A). Constant neutral loss scan of M8 (B). Proposed structural formulas of M8 and corresponding MS² fragments (C).

1.2.2. Identification of the M9 metabolite of INF

The M9 metabolite appeared at 21.8 min and had a MIP at *m/z* 608, which was 80 Da higher than that of the precursor ion (M5) with an *m/z* 528 that is proposed to be a sulfate conjugation metabolite of INF[38]. CID of MIP at *m/z* 608 generated fragment ion at *m/z* 528, *m/z* 591 and *m/z* 415 (Fig. S9A). Fragment ion at *m/z* 528 was 80 Da less than the precursor ion, suggesting the loss of a sulphate group from the benzene ring in part A of the INF structure. The daughter ion *m/z* 591 indicated the loss of a hydroxyl group from the benezen ring in part A of the INF structure, which matched the other fragment ions at *m/z* 415 (Fig. S9C). Another LC-MS/MS screening for sulfate adducts was performed by constant neutral loss scan monitoring for ions that lose 80 Da[39] (Fig. S9B).

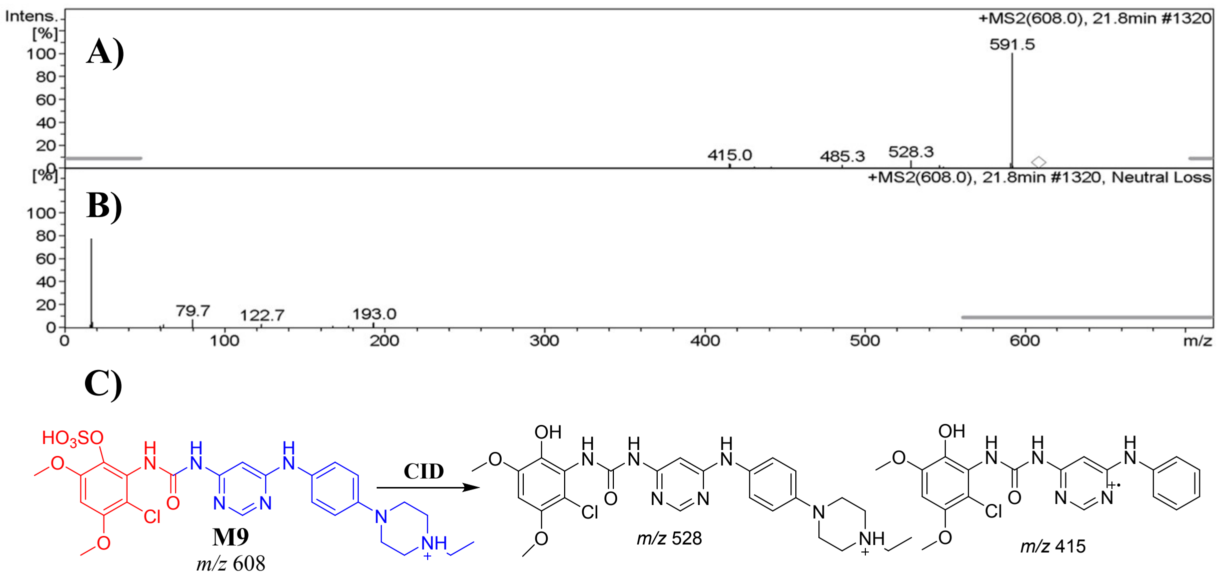

Fig. S9. Positive ion MS^2 mass spectrum of M9 21.8 min. (A). Constant neutral loss scan of M9 (B). Proposed structural formulas of M9 and its corresponding MS^2 fragments (C).

1.2.3. Identification of the INF M10 metabolite

The INF M10 metabolite appeared at 24.3 min. and had MIP at *m/z 722*, which was 176 Da higher than that of the precursor ion with *m/z* 546 (M2) proposed to be a glucuronic acid conjugate metabolite of INF. CID of MIP at *m/z* 722 generated fragment ion at *m/z* 546 (Fig. S10A) and was 176 Da less than the precursor ion, suggesting the loss of the glucuronic acid conjugate that matched the other fragment ions at *m/z* 313 (Fig. S10C). Another LC-MS/MS screening for glucuronic acid conjugate was performed by Constant neutral loss scans for M10 metabolite conjugates were more specific due to the characteristic loss of 176 mass units, and provided much cleaner mass spectral data [38] (Fig. S10B).

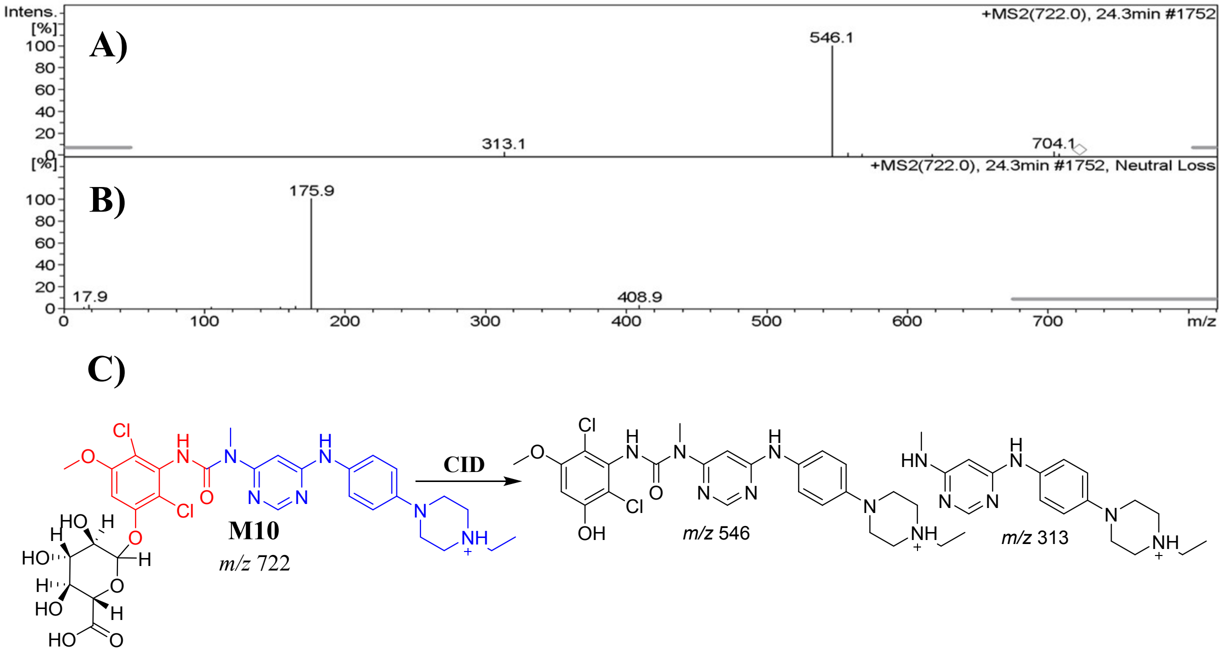

Fig. S10. Positive ion MS^2 mass spectrum of M10 at 24.3 min. (A). Constant neutral loss scan of M10 (B). Proposed structural formulas of M 10 and corresponding MS^2 fragments (C).

1.3. Identification of *in vivo* phase I metabolites of INF

1.3.1. Identification of the M20 metabolite of INF

The M20 PIPs appeared at 30.8 min. The M20 was the net product of double hydroxylation metabolite of INF. The CID of M19 at *m/z* 592 gave five daughter ions at *m/z m/z* 575, *m/z* 345, *m/z* 328, *m/z* 298 and *m/z* 221 (Fig. S11A). The production of the daughter ion at *m/z* suggested that there was double hydroxyl group (+32) added onto the piperazine moiety (part b) which matched with the daughter ions at *m/z* 345, 328 and 298 (Fig. S11B).

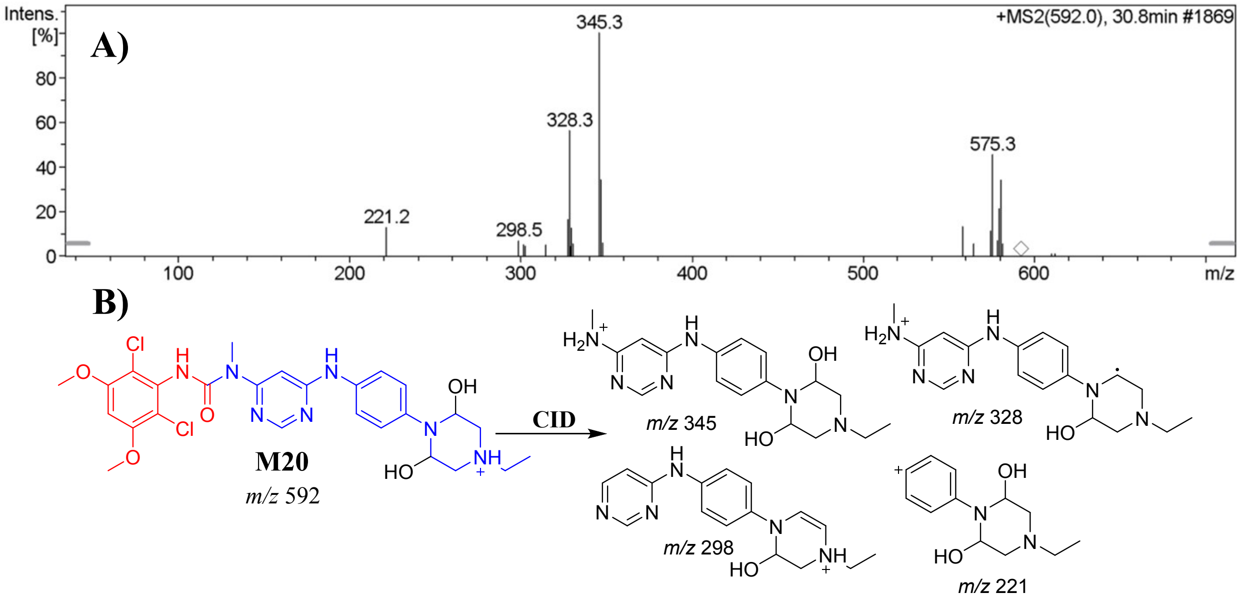

Fig. S11. Positive ion MS^2 mass spectrum of M20 at 30.8 min. (A). Proposed structural formulas of M20 and corresponding MS^2 fragments (B).

1.3.2. Identification of the M21 metabolite of INF

The M21 PIPs appeared at 27.4 min. The M21 was the net product of *N*-dealkylation, *N*-demethylation and *O*-demethylation of two methyl groups. The CID of M20 at *m/z* 490 gave daughter ions at 472, *m/z* 314 and *m/z* 163 (Fig. S12A). The fragment ions at *m/z* 163 and *m/z* 314 suggested that the metabolic pathway included N-dealkylation of the N-ethyl piperazine ring, *N*-demethylation of the nitrogen atom attached to pyrimidine group and *O*-demethylation of two methyl groups that matched the other fragment ions at *m/z* 472 (Fig. S12B).

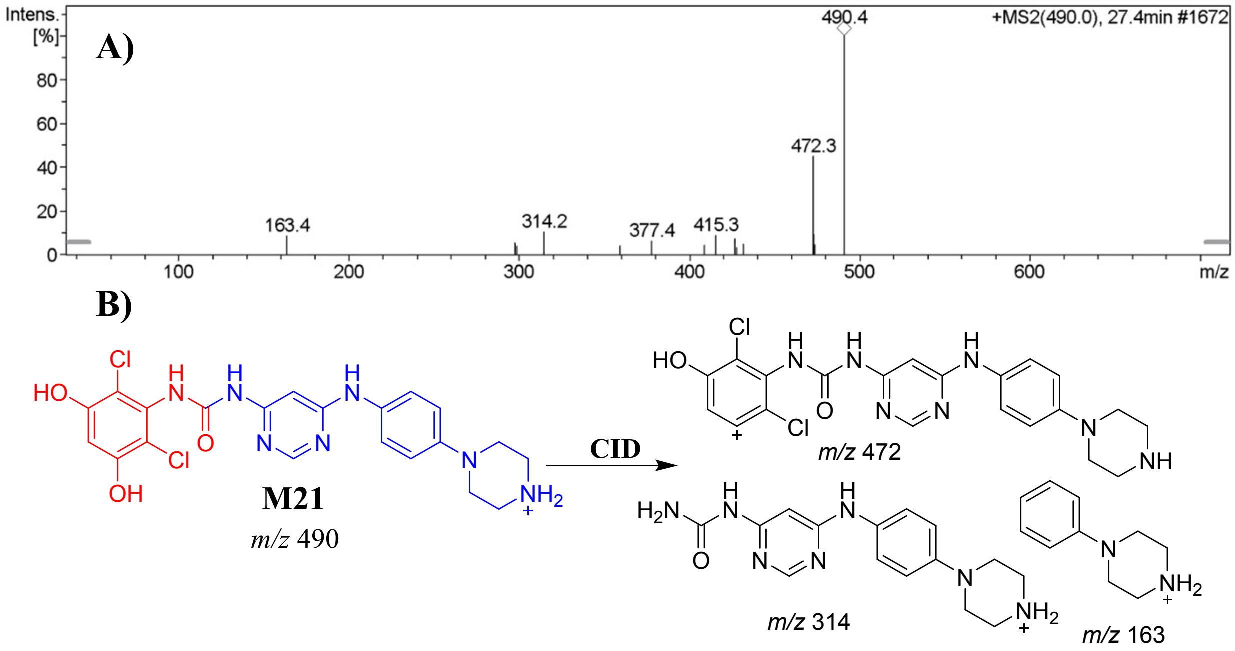

Fig. S12. Positive ion MS^2 mass spectrum of M21 at 27.4 min. (A). Proposed structural formulas of M21 and corresponding MS^2 fragments (B).

1.4. Identification of the in vivo phase II metabolites of INF

1.4.1. Identification of the M22 metabolite of INF

The M22 appeared at 29.5 min. and had MIP at *m/z 752*, which was 176 Da higher than that of the precursor ion with *m/z* 546 (M2) proposed to be a glucuronic acid conjugate metabolite of INF. CID of MIP at *m/z* 752 generated fragment ion at at *m/z* 576 (Fig. S13A) and was 176 Da less than the precursor ion, suggesting the loss of the glucuronic acid conjugate that matched the other fragment ions at *m/z* 291 and *m/z* 505 (Fig S13C). Another LC-MS/MS screening for glucuronic acid conjugate was performed by Constant neutral loss scans for M21 metabolite conjugates were more specific due to the characteristic loss of 176 mass units, and provided much cleaner mass spectral data [38] (Fig. S13B).

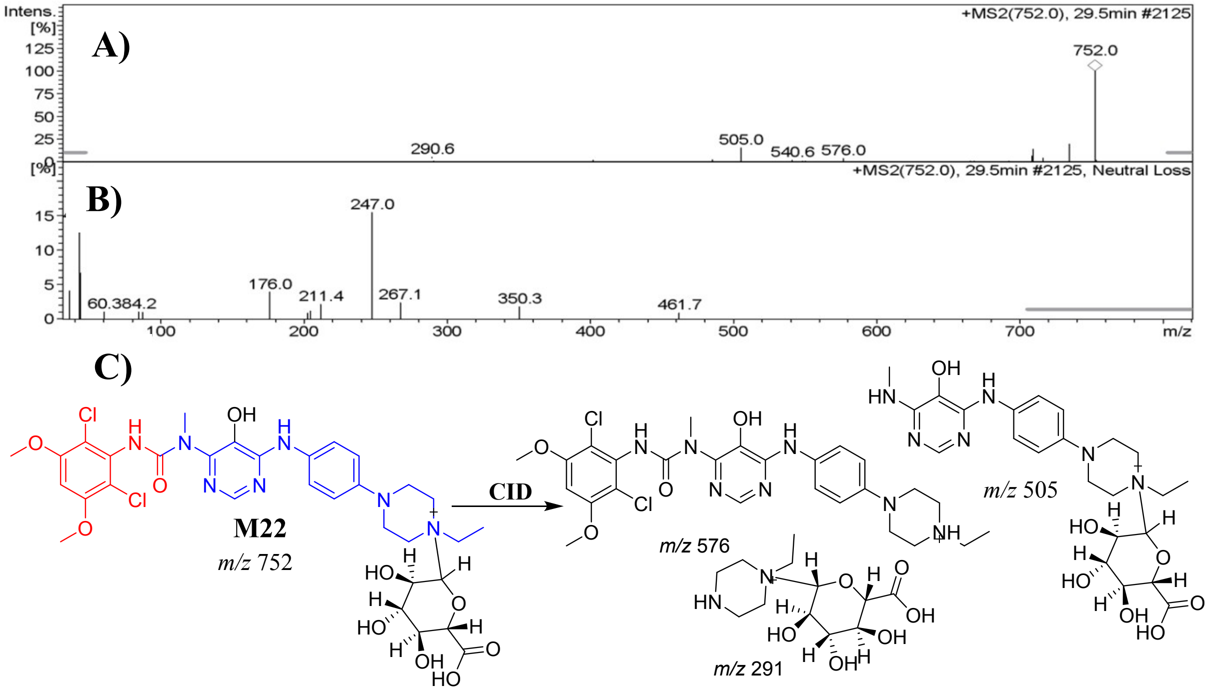

Fig. S13. Positive ion MS^2 mass spectrum of M22 at 29.5 min. (A). Constant neutral loss scan of M22(B). Proposed structural formulas of M22and its corresponding MS^2 fragments (C).
